# Unique behavior of *Trypanosoma cruzi* mevalonate kinase: A conserved glycosomal enzyme involved in host cell invasion and signaling

**DOI:** 10.1038/srep24610

**Published:** 2016-04-26

**Authors:** Éden Ramalho Ferreira, Eduardo Horjales, Alexis Bonfim-Melo, Cristian Cortez, Claudio Vieira da Silva, Michel De Groote, Tiago José Paschoal Sobreira, Mário Costa Cruz, Fabio Mitsuo Lima, Esteban Mauricio Cordero, Nobuko Yoshida, José Franco da Silveira, Renato Arruda Mortara, Diana Bahia

**Affiliations:** 1 Departamento de Microbiologia, Imunologia e Parasitologia, Escola Paulista de Medicina, Universidade Federal de São Paulo, São Paulo, SP, Brazil; 2 Instituto de Física, USP, São Carlos, São Carlos, SP, Brazil; 3 Instituto de Ciências Biomédicas, Universidade Federal de Uberlândia, Uberlândia, MG, Brazil; 4 Laboratório Nacional de Biociências, Campinas, SP, Brazil; 5 Departamento de Biologia Geral, Instituto de Ciências Biológicas, Universidade Federal de Minas Gerais, MG, Brazil

## Abstract

Mevalonate kinase (MVK) is an essential enzyme acting in early steps of sterol isoprenoids biosynthesis, such as cholesterol in humans or ergosterol in trypanosomatids. MVK is conserved from bacteria to mammals, and localizes to glycosomes in trypanosomatids. During the course of *T. cruzi* MVK characterization, we found that, in addition to glycosomes, this enzyme may be secreted and modulate cell invasion. To evaluate the role of TcMVK in parasite-host cell interactions, TcMVK recombinant protein was produced and anti-TcMVK antibodies were raised in mice. TcMVK protein was detected in the supernatant of cultures of metacyclic trypomastigotes (MTs) and extracellular amastigotes (EAs) by Western blot analysis, confirming its secretion into extracellular medium. Recombinant TcMVK bound in a non-saturable dose-dependent manner to HeLa cells and positively modulated internalization of *T. cruzi* EAs but inhibited invasion by MTs. In HeLa cells, TcMVK induced phosphorylation of MAPK pathway components and proteins related to actin cytoskeleton modifications. We hypothesized that TcMVK is a bifunctional enzyme that in addition to playing a classical role in isoprenoid synthesis in glycosomes, it is secreted and may modulate host cell signaling required for *T. cruzi* invasion.

*Trypanosoma cruzi* is the etiological agent of Chagas’ disease or American trypanosomiasis, a prevalent health problem that affects 6–7 million people worldwide, mostly in Latin America^1^. *T. cruzi* has a complex life cycle involving invertebrate and vertebrate hosts. Trypomastigotes and extracellular amastigotes are the infective forms for mammalian hosts and they may engage a variety of strategies to infect and survive in mammalian host cells^2^,^3^.

*T. cruzi* does not synthesize cholesterol *de novo*, but instead synthesizes ergosterol and enzymes of sterol biosynthesis pathway could be potential targets for development of anti-trypanosomal drugs^4^. Mevalonate kinase (MVK) is an important enzyme of the isoprenoid/cholesterol biosynthesis pathway, catalyzing the phosphorylation of mevalonic acid into phosphomevalonate^5^. Mevalonate kinases are found in a wide variety of organisms from bacteria to mammals. The mevalonate pathway provides cells with essential bioactive molecules vital in multiple cellular processes^6^ via conversion of mevalonate into sterol isoprenoids including cholesterol, an indispensable precursor of lipoproteins and steroid hormones, a number of hydrophobic molecules and nonsterol isoprenoids. These intermediates of the mevalonate biosynthetic pathway play important roles in the post-translational modification of a variety of proteins involved in the intracellular signaling essential in cell growth/differentiation, gene expression, protein glycosylation and cytoskeletal assembly^6^,^7^.

Recently, it has been demonstrated that in the trypanosomatids *Trypanosoma brucei* and *Leishmania major* sterol biosynthesis is distributed in multiple intracellular compartments and the production of HMG-CoA from acetyl Coenzyme A and generation of mevalonate mainly occurs in the mitochondrion while further mevalonate phosphorylation is almost exclusively located in glycosomes^8^. During the course of the characterization of *T. cruzi* MVK (TcMVK), we found that, in addition to glycosomes, this enzyme may be secreted and modulate cell invasion, possibly by phosphorylation of host cell components. Given this unexpected behavior we hypothesized that TcMVK may be a bifunctional enzyme, typified by having a second function not related to its original, classical function. In addition, many enzymes that are originally involved in metabolic pathways can also act as virulence factors of pathogenic protozoa^9^,^10^,^11^,^12^. The present study demonstrates a new role for a metabolic enzyme of *T. cruzi.*

## Materials and Methods

### Ethics Statement

All experiments involving animal work were conducted under Brazilian National Committee on Ethics Research (CONEP) ethic guidelines, which are in accordance with international standards (CIOMS/OMS, 1985). The protocol was approved by the Committee on Ethics of Animal Experiments of Universidade Federal de São Paulo (Permit Number: CEP 0913/10). During the experimental procedures, all efforts were made to minimize animal suffering.

### Parasites and mammalian cells

*T. cruzi* isolates used in this study were: strain CL and clone CL Brener (DTU VI^13^,^14^), and strain G (DTU I^13^,^15^). Extracellular amastigotes (EAs) were obtained by differentiation of tissue culture trypomastigotes (TCTs) in LIT medium at pH 5.8 for 14 h as previously described^16^. Epimastigotes (EPs) and metacyclic trypomastigotes (MTs) were obtained as previously described^17^. HeLa cells (Instituto Adolfo Lutz, São Paulo, SP, Brazil) were grown in RPMI 1640 medium (Sigma-Aldrich, St Louis, MO, USA) supplemented with 10% fetal bovine serum (FBS, Invitrogen, Carlsbad, CA, USA), 10 μg/mL streptomycin, 100 U/mL penicillin and 40 μg/mL gentamycin at 37 °C and 5% CO_2_.

### Invasion assays

HeLa cell invasion assays were performed by adding 500 μL of cell suspension (1.5 × 10^5^) to 24 well plates containing sterile glass coverslips and incubated overnight at 37 °C and 5% CO_2_. Cells were treated with 300 nM of rTcMVK in RPMI/10% FBS and incubated at 37 °C in a CO_2_ (5%) humidified incubator for 1 h and washed twice with sterile PBS. Next, parasite suspensions at 10:1 per cell for EAs and 20:1 for MTs were added and the plates incubated for another 2 h at 37 °C in a 5% CO_2_ humidified incubator. The cells were then gently washed three times with PBS to remove unattached parasites, fixed with Bouin and stained with Giemsa as previously described^18^. For antibody inhibition assays parasites were incubated for 30 min with anti-MVK, specificity control α-C03^19^, rTcMVK or untreated, washed then incubated with HeLa cells as described above.

### Cloning of TcMVK alleles

Alleles were amplified by PCR from genomic DNA of clone CL Brener, G and CL strains and cloned into pGEM^®^-T Easy Vector (Promega, Fitchburg, WI, USA). The coding sequences of TcMVK were amplified using the following primers: XM_797435.1 (Esmeraldo like), forward- 5′ CTA AAT TTT GGC ACT TCT AGG GCA 3′ and Reverse: GAA GTA CAG GAA CGT TAT TTA ACC T; and XM_809535.1 (non-Esmeraldo like), forward *BamHI*-5′-GGC CGG GGA TCC GAG CGA ACA GAG AAG AAC C 3′ and reverse *HindIII*-5′-GGC CGG AAG CTT AGG CAC TTC TAG GGC ACG CAG 3. The recombinant clones were sequenced by using the dideoxynucleotide chain-termination method^20^ according to manufacturer’s protocol. Gene sequencing was performed in an automatic ABI PRISM 3100 sequencer (Applied Biosystem, Foster City, CA, USA) using the primers above and internal primers for TcMVK (internal: GTT CAC TTC ATC TTC GGT CA) and TcKMV2 (3′ internal primer forward 1: CGT CCT GCT GTGCCAGG and internal primer forward 2: CGG CCG CGA CAT TTG GT). Sequence edition and analyses were performed using DNASTAR Lasergene Editseq ( www.dnastar.com).

### Expression of the recombinant protein TcMVK (rTcMVK)

TcMVK was cloned in fusion with amino terminal His6 tag in plasmid pET-28a (Merck, Darmstadt, Germany). In order to amplify the fragment from G strain genomic DNA, the following primers were used: forward *BamHI*-5′-GGC CGG GGA TCC GAG CGA ACA GAG AAG AAC C 3′ and reverse *HindIII*-5′-GGC CGG AAG CTT AGG CAC TTC TAG GGC ACG CAG 3′. Amplification was performed using *Pfu* ultra II DNA polymerase (Agilent, Santa Clara, CA, USA) in a final volume of 50 μL. PCR conditions were: 35 cycles of 1 min at 94 °C, 1 min at 94 °C, 30 s at 50 °C, 1 min at 72 °C, and a final extension of 10 min at 72 °C. *E. coli* BL21 strain cells were transformed with pET-28a:TcMVK plasmid and grown at 20 °C for 48 h, 150 rpm, in 4 L ZYM-5052 high induction medium^21^ containing kanamycin (30 μg/mL). Cells were harvested by centrifugation (40 min, 6000 g), the pellet re-suspended in one tenth of culture volume in buffer A (50 mM Tris, 250 mM NaCl, 20 mM imidazole, pH 7.5) and stored at −20 °C.

### Purification of rTcMVK

Cells were incubated with lysozyme (100 μM) in an ice bath for 40 min and then were lysed by sonication using a Branson Sonifier 450 (VWR Scientific, Radnor PA, USA) equipped with a 2 mm-diameter tip. Debris was removed by centrifugation (60 min, 4500 g) and the clear supernatant used for protein purification. Prior to size exclusion chromatography the protein was dialyzed in glycine buffer pH 9.0 (30 mM), NaCl (50 mM). The Ni-NTA Superflow (Qiagen, Venlo, Germany) column was washed with MilliQ water, equilibrated with start buffer A and the cleared sample applied to the Ni-NTA Superflow column (Qiagen, Venlo, Germany). The column was washed with ten column volumes of start buffer A, followed by 5 volumes of elution buffer (buffer A with 230 mM imidazole). Samples with high OD_280_ were collected and concentrated to an OD_280_ of 5.0 AU (nanoDrop Thermo, Waltham, MA, USA) and the gel filtration step was performed using Superdex 200 High Load 16/60 prep-grade with glycine buffer pH 9.0 containing 0.15 M NaCl, which permits size separation of MVK monomers and dimers. The fractions corresponding to the rTcMVK peak were diluted five times in glycine buffer pH 9.0 without NaCl, concentrated and used for activity measurements. Purified rTcMVK was visualized in SDS-PAGE gels (Figure SM5). Purified rTcMVK was stored at 4 °C, 1 mg/mL.

### Binding of rTcMVK to HeLa cells

5 × 10^4^ HeLa cells/well were seeded in 96 well microplates and grown at 37 °C for 24 h. Subsequently, cells were fixed with 3.5% paraformaldehyde in PBS and blocked with 10% FBS in PBS for 1 h at room temperature (RT). Cells were incubated with increasing amounts of rTcMVK or rEnolase used as a negative control for 1 h^22^. Subsequent steps were performed as previously described^22^.

### Production of anti-TcMVK antibodies

Anti-TcMVK polyclonal antibodies were obtained by subcutaneous immunization of BALB/c mice with purified rTcMVK in association with complete or incomplete Freund’s adjuvant (Sigma-Aldrich, St. Louis, CA, USA). Mice immunization was performed administering four 100 μg doses of rTcMVK at 15 day intervals. Serum was collected and titrated to 1:3200 by an enzyme-linked immunosorbent assay (ELISA) with purified rTcMVK. Western blots using total G extracts were performed to verify antibody specificity (Figure SM4).

### Detection of TcMVK into supernatants of *T. cruzi* cultures

To obtain the parasite conditioned medium, EAs and MTs were washed in serum free RPMI and incubated in the same medium at a concentration of 1 × 10^8^ cells/mL for 16 h at 37 °C as previously described^22^. Following 16 h incubation, parasites were removed by centrifugation at 3000 *g* for 10 min at 4 °C. Parasite-free supernatants were filtered with 0.45 μm-pore-size filters (Millipore) and concentrated by trichloroacetic acid (TCA) precipitation, submitted to 13% SDS-PAGE and visualized by Western blot using anti-TcMVK at a dilution of 1:1000. Parasite viability was determined by propidium iodide incorporation at the beginning and end of the experiment and approximately 96% of cells were viable as determined in both measurements.

### TcMVK kinase activity

Specific rTcMVK activity was measured as previously described^23^ with minor modifications. Briefly, a stock standard solution of 0.1 M (RS)-mevalonic acid was prepared by dissolving 65 mg of crystalline mevalonic acid lactone (Sigma-Aldrich, St. Louis, MO, USA) in 3 mL of 0.2 N potassium hydroxide (KOH) and heated at 37 °C for 1 h to hydrolyze the lactone. The pH was adjusted to 7.2 with 0.1 N hydrochloric acid (HCl) and the volume brought to 5 mL with water. Measurement of enzyme activity was carried out at 25 °C on a Spectra max Plus 384, 96 well plate, UV/V spectrometer (Molecular Devices, Sunnyvale, CA, USA). In accordance with the manufacturer’s instructions, 0.2 mL of mix solution containing glycine buffer (100 mM) pH 9.0, NaCl (25 mM), Lactic dehydrogenase (4 U), Pyruvate kinase (4 U), mevalonate^*^ (4 mM), β-NADH (30 μM), ATP (5 mM), Magnesium Chloride (5 mM), Phosphoenolpyruvate (1 mM), rTcMVK (50 ng to 2500 ng). The enzyme rTcMVK was used to start the reaction. OD_340_ was monitored for 10 min, recorded every 20 seconds. One unit of enzyme activity was defined as the amount of activity required to produce 1 µmol of mevalonate 5-phosphate (measured as µmol of NADH consumed) per minute per mg of enzyme. Blank assays were performed in the absence of ATP. All the measurements were repeated at least three times.

### Immunofluorescence assays

Double immunofluorescence assays were performed with anti-TcMVK, anti-aldolase (glycosomal marker, raised in rabbit) and anti-binding immunoglobulin protein (BiP) [endoplasmic reticulum (ER) marker, raised in rabbit], provided by Dr. Sergio Schenkman, UNIFESP. Developmental forms (EPs, MTs and EAs) of the G strain were washed with PBS and fixed with 3.5% paraformaldehyde in PBS for 15 min at RT, washed with PBS in 1.5 mL microcentrifuge tubes, then incubated with blocking/permeabilizing solution PGS (0.2% gelatin, 0.1% saponin and 0.1% NaN_3_, diluted in PBS) for 1 h at RT. Cells were then washed with PBS and incubated with primary antibodies diluted 1:50 in PGS for 24 h at 4 °C, washed with PBS and incubated with secondary antibodies for 1 h at RT: anti-mouse Alexa 568 (Invitrogen, Carlsbad, CA, USA), or anti-rabbit Alexa 488 (Invitrogen, Carlsbad, CA, USA) diluted 1:200 in PGS containing 1 μg/mL 4′6,-diamidino-2-phenylindol (DAPI, Sigma-Aldrich, St. Louis, MO, USA) to label nuclei and kinetoplasts. Parasites were then washed with PBS and mounted in glycerol buffered with 0.1 M Tris, pH 8.6, with 0.1% *p*-phenylenediamine as anti-fade agent. Images were acquired with a TCS SP5 II Tandem Scanner confocal microscope (Leica Microsystems, Wetzlar, Germany) using a 100× NA 1.44 PlanApo oil immersion objective and processed with Imaris^®^ (Bitplane).

### Phosphoprotein assays and Western blotting

HeLa cells (5 × 10^6^) were seeded onto 10 cm (diameter) plates and grown for 24 h. Cells were incubated with serum-free RPMI for another 24 h (starvation) to decrease cell constitutive signaling. Following starvation, cells were incubated with rTcMVK or parasites (EAs and MTs) for the following time periods: 0 (without contact), 1, 5, 15, 30, 60, 90 and 120 min. Following incubation, cells were washed with PBS and removed from the plates with a cell scraper in a solution of cold PBS (4 °C) containing 2 mM Na_3_VO_4_ and NaF to inhibit intrinsic phosphatase activity. Cells were then centrifuged and lysed with mammalian cell lysis buffer (mPER, Thermo, Waltham, MA, USA) containing 5 mM Na_3_VO_4_ and 2 mM NaF. Protein quantification was performed using the BCA kit (Thermo, Waltham, MA, USA) according to the manufacturer’s instructions. Subsequently, samples were submitted to 10% SDS-PAGE, transferred to PVDF membranes and blocked with skimmed milk (Cell Signaling, Danvers, MA, USA). Antibodies to different phosphoproteins were diluted according to the manufacturer´s instructions. Membrane blocking and primary and secondary antibodies (conjugated with peroxidase) incubations were performed using Na_3_VO_4_ 250 nM in Tris-buffered saline (TBS). Bound antibodies were amplified with ECL (GE Healthcare Little Chalfont, UK) and luminescent bands visualized using the UVITEC photo documenter (UVItec. Cambridge, UK). Band densitometry was performed using UVIBAND software. The antibodies used were specific to phosphorylation residues implicated in enzyme activation, namely: Src family kinases (pSFKs), Y416; pFAK, Y397; pERK1/2, T202/Y204; p-p38 T180, Y182 (Cell Signaling, Danvers, MA, USA), pPAK, S144 (Invitrogen, Carlsbad, CA, USA) and mouse anti-actin (A2228, Sigma-Aldrich, St. Louis, MO, USA) used as a loading control.

### Protein prediction, alignment and similarities search

Motif scanning within the predicted MVK from the *T. cruzi* genomic database was performed in the ExPASy proteomics server ( http://www.expasy.org). Primary structure predictions were constructed by the on-line program SMART^24^. Alignment of TcMVK with MVK of other trypanosomatids was performed using ClustalW^25^.

### Molecular homology, dynamics and structural analyses

The TcMVK structure was homology modeled, with YASARA Software ( www.yasara.org), based on two *Leishmania major* MVK structures (2HFU and 2HFS). Preparation of TcMVK structure for dynamics began by adding hydrogen atoms. Proteins were then subsequently added in a water box and charges neutralized by adding sodium and chlorine atoms. Initially the system was submitted to solvent energy minimization followed by protein energy minimization and, finally, total system energy minimization. After stabilization, the system was submitted to molecular dynamics for 30 ns at 298 K for stability evaluation measured via the root-mean-square deviation (RMSD) value in Angstroms (Å). High RMSD values were considered indicative of instability or high variation of atom position that may result in model dissociation. Molecular dynamics results were visually examined using the VMD program (Visual Molecular Dynamics). The volume of catalytic pocket was measured using KVFinder^26^, and the volume of the mevalonic acid was measured using UCSF Chimera^27^.

### Statistical analysis

Statistical analysis of invasion assays was performed with GraphPad Prism employing Student’s t test. Data are present as mean standard deviation (SD). *P < 0.05 and ***P < 0.001 mean significance.

## Results

### Molecular characterization of *T. cruzi* MVK

In *T. cruzi* (clone CL Brener) genome there are two annotated TcMVK genes (XM_797435 and XM_809535) which differ from each other by the presence, in the large TcMVK variant (XM_797345), of a 5′ terminal extension containing two in frame putative ATG initiator codons (Figure SM1). The region immediately after the first ATG (on XM_797345) encodes a predicted 19-amino acid signal peptide that is missing from the short TcMVK copy (XM_809535) (Figure SM1). The nucleotide sequence encoding the putative signal peptide lies in a polypyrimidine tract followed by an AG trans-splicing acceptor dinucleotide suggesting that this region could correspond to the 5′-UTR of the TcMVK gene. In fact, this trans-splicing acceptor site is also present in the intergenic region of the copy encoding the short TcMVK variant (Figure SM1, sequence XM_809535 extent). To investigate more carefully this possibility, we performed RT-PCRs on mRNA of CL Brener epimastigotes (EPs) using an internal TcMVK reverse oligonucleotide and a mini-exon as forward oligonucleotide.

Analysis of ten cDNA clones revealed that most of the MVK mRNAs (n = 9) start immediately after an alternative AG splicing acceptor site which is only present in the intergenic region of the copy encoding the short MVK variant (Figure SM1, cDNA clones E500A and E500B). This alternative AG is located 14 nt upstream from that indicated in the XM_797435. We found only one cDNA clone (Figure SM1, clone E500C) starting immediately after the AG splicing-acceptor site of the large TcMVK (XM_797435) indicating that the polypyrimidine-rich region encoding the putative signal peptide is indeed pointing out the intergenic region that will undergo trans-splicing reaction. Given that this mapped AG is common to both the large and short copies of TcMVK we cannot rule out an alternative splicing event on the short TcMVK copy. The results suggest a preferential transcription of TcMVK lacking the signal peptide either from the large or short TcMVK copies. Differences in the TcMVK large/short copy ratio could be due to a bias during the RT-PCR or cloning process. Analysis of a larger number of clones would be necessary to estimate the TcMVK copies ratio more precisely. The nucleotides on the predicted CDS of both copies of TcMVK amplified by RT-PCR are 99% identical with only six differences and no gaps.

Finally, analysis on NCBI’s SRA database containing sequence data from next-generation sequencing experiments allowed us to identify only one read of TcMVK containing the spliced-leader in poly(A)^+^ selected mRNAs isolated from metacyclic trypomastigotes (MTs) of *T. cruzi* Dm28c. The read (SRR1587296.10777788.1) mapped on the AG splicing-acceptor site of the large TcMVK (XM_797435). Taken together, the above results offer strong evidence that the predicted signal peptide of MVK is indeed an artifact derived from the algorithm used to predict the CDS from a genomic sequence.

**Figure 1:**
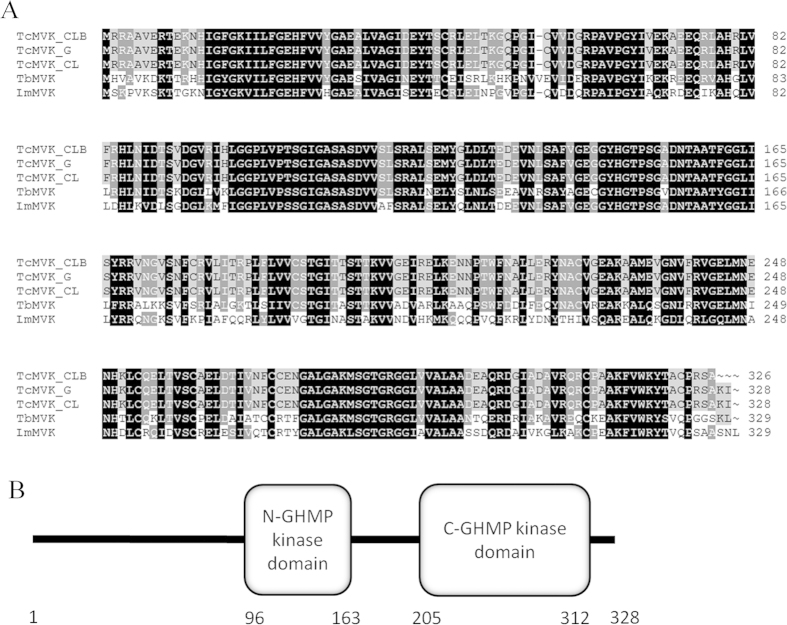
TcMVK is similar to MVK from other trypanosomatids. (**A**) Multiple sequencing alignment demonstrates that TcMVK is approximately 60% similar to MVK from other trypanosomatids. Tc: *Trypanosoma cruzi*: CLB: clone CL Brener (XP_802528); G: G strain; CL: CL strain; Tb: *Trypanosoma brucei brucei* (XP_844 557, 64% identity); Lm: *Leishmania major* (XP_001685041, 58% identity). Shade code: Black, 100% identity; dark gray, 80% identity; light gray, 60% identity. (**B**) Schematic drawing of the primary structure of TcMVK proteins from *T. cruzi* clone CL Brener generated with SMART^24^. Similar to other MVKs, TcMVK presents two kinase domain (blast.ncbi.nlm.nih.gov).

The presence of TcMVK genes in clone CL Brener was experimentally confirmed by genomic PCR amplification. Sequences from G (GenBank KR350584) and CL (GenBank KR350585) strains were obtained with the same approach and compared to that of CL Brener showing that MVK is conserved across these strains (Fig. 1A). The alignment of TcMVK with those of other trypanosomatids showed that MVK is conserved among trypanosomatid species (identity of 64% and 58% with *Trypanosoma brucei* and *Leishmania major*, respectively) (Fig. 1A). Similar to other organisms *T. cruzi* MVK protein contains two GHMP kinase domains most likely involved in ATP binding (Fig. 1B).

### Structure of TcMVK protein

**Figure 2:**
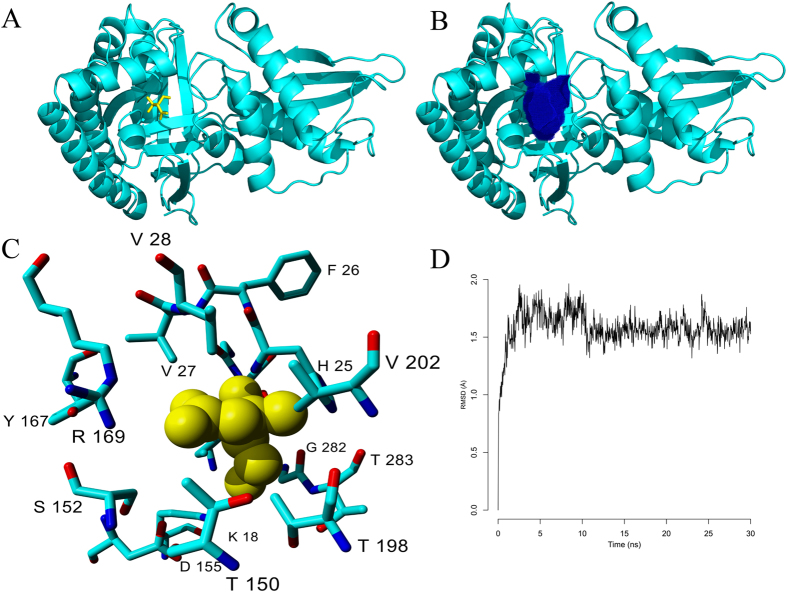
TcMVK homology modeling and structural analysis. (**A**) TcMVK cartoon representation (cyan) and mevalonic acid (yellow). (**B**) TcMVK cartoon representation (cyan) and catalytic pocked (blue). (**C**) Amino acids predicted to be involved in mevalonic acid binding and their positions in relation to mevalonic acid. The interaction model was generated based on similarities with the crystal structure of *L. major* MVK bound to mevalonic acid (yellow). (**D**) Molecular dynamics analysis of the TcMVK proposed model generated by YASARA ( www.yasara.org). The carbon alpha RMSD in angstroms (Å, Y axis) is presented in relation to time (ns, X axis).

The TcMVK tridimensional structure was modeled by YASARA. The best model was created based on the *Leishmania major* MVK crystals, PDB ID: 2HFS and 2HFU. The TcMVK amino acid sequence has 58% of identity with both structures. The best model is shown in Fig. 2A; in yellow the predicted position of mevalonic acid in the catalytic pocket. Figure 2B shows the predicted region of the catalytic pocket in blue. The pocket volume is 340 Å^3^ and mevalonic acid volume is 119 Å^3^. Figure 2C shows the amino acids likely to be involved in mevalonic acid binding based on their positions in relation to the substrate conformation. Figure 2D shows the result of molecular dynamics of TcMVK model performed by YASARA ( www.yasara.org). This analysis shows a small destabilization at the first 5 ns followed by stabilization at the following 25 ns indicating that the proposed 3D structure is viable.

### The dimeric, not monomeric, TcMVK fraction has a high enzymatic activity

The gene assigned encoding a putative TcMKV was amplified by PCR, cloned into *E. coli* and expressed as a recombinant protein (rTcMVK) tagged with poly-histidine (Figure SM5). The *E. coli* strain over-expressing rTcMVK presented a slow growth and low enzyme over-expression in most of the commonly used culture media. The use of the medium ZYM5052 resulted in a very high increase of the expression and a high yield of pure protein (in relation to other media), with a final amount of 15 mg of protein, in 1L of expression medium. After the size exclusion chromatography to separate the oligomerization states we obtained three peaks with molecular weight corresponding to tetramer (a), dimer (b) and monomer (c) (Figure SM6A).

The monomeric samples, fresh collected, presented very low activity as compared with the dimeric fraction (fresh) (Figure SM6B). Even more, after 15 days stock of the monomeric enzyme, a new run on the gel filtration column has shown that more than 60% of the protein moved to large aggregates or dimeric form (less than 20%). We measured the specific activity of dimeric state resulting in 74 μM/(min.mg) (SM6 B and Table 1).

Thus, we have concluded that the monomeric samples have not shown any catalytic activity (SM6 B), as already observed by Sgraja *et al*.^28^ for *Leishmania* and *T. brucei* recombinant enzymes^28^. Moreover, the dimeric samples have shown a high enzymatic activity over the sensibility of the method and comparable to other known species (Table 1).

#### Expression and subcellular distribution of TcMVK

**Figure 3:**
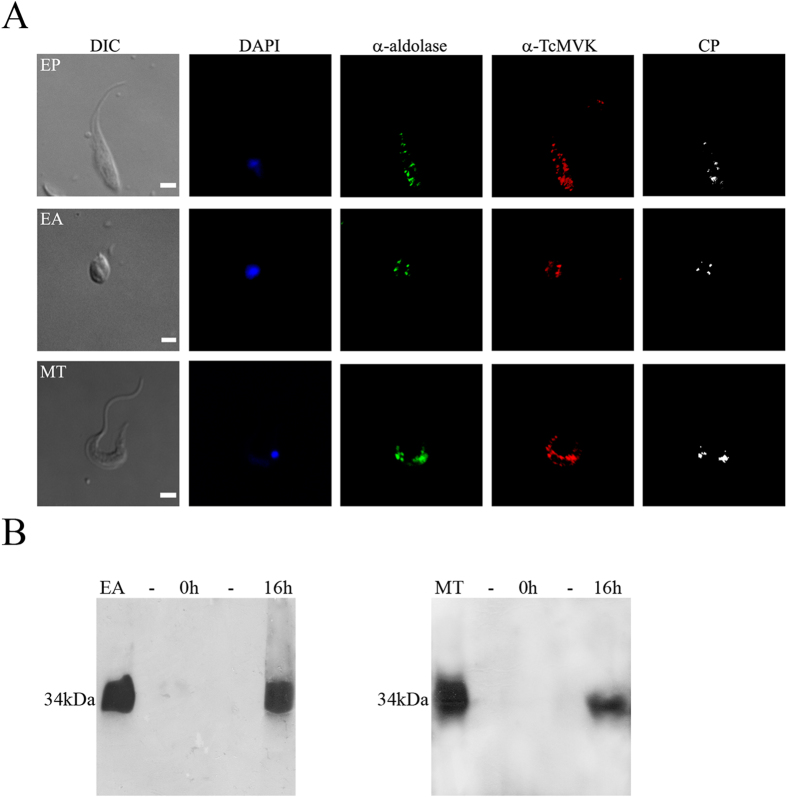
Intracellular distribution of TcMVK. (**A**) TcMVK partially co-localized with aldolase. Immunofluorescence images acquired with confocal microscope show that TcMVK is localized in glycosomes of *T. cruzi* epimastigotes (EP), extracellular amastigotes (EA) and metacyclic trypomastigotes (MT). Differential interference contrast (DIC); DAPI (blue); rabbit anti-aldolase (green); mouse anti-TcMVK (red) and colocalized pixels (CP, white). Bar: 4 μm. Images are representative of three independent experiments. (**B**) Western blot analysis of EA (left panel) and MT (right panel) conditioned medium confirming secretion of TcMVK; EAs and MTs were incubated for 16 h in RPMI at 37 °C (1 × 10^8^ parasites/ml). After centrifugation, the supernatant from 1 × 10^8^ parasites was precipitated with trichloroacetic acid and subjected to SDS-PAGE (13% gel). The cell pellet was solubilized in Laemmli buffer and the equivalent of 1 × 10^8^ parasites was submitted to SDS-PAGE (13%). Anti-MVK was raised in mice using recombinant TcMVK and used at a dilution of 1:1000. EA and MT: whole cellular lysate (centrifugation pellet) from extracellular amastigotes and metacyclic trypomastigotes respectively; 0 h: Incubation time point zero; 16 h: Extracellular amastigotes or metacyclic trypomastigotes 16 h conditioned medium; (−): Empty lanes; 34 kDa: TcMVK expected molecular mass. These results are representative of two independent experiments.

The TcMVK of all *T. cruzi* forms is found in glycosomes, as deduced from its partial colocalization with aldolase (Fig. 3A). Additional immunofluorescence experiments with endoplasmic reticulum, acidic compartments or mitochondrial markers indicated no colocalization with TcMVK (Figure SM7). Expression of *T. cruzi* native TcMVK proteins was also analyzed by subjecting EAs and MTs whole extracts to SDS-PAGE and immunoblotting analysis. Anti-TcMVK antibodies reacted with a 34 kDa protein in both developmental forms, in agreement with the predicted molecular mass of 34 kDa (Fig. 3B). To investigate whether TcMVK is secreted into the extracellular medium, EA and MT forms were incubated overnight in RPMI medium and the supernatants collected. Western blot analysis of secreted products in conditioned medium carried out with anti-TcMVK antibodies showed that *T. cruzi* EAs and MTs secreted 34 kDa-TcMVK (Fig. 3B left and right panel respectively).

#### Recombinant TcMVK affects parasite invasion of HeLa cells

**Figure 4:**
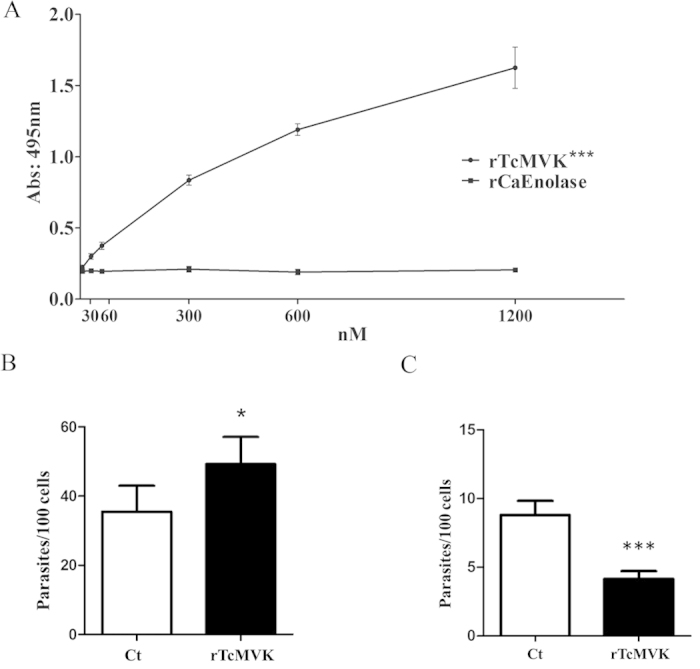
rTcMVK adheres to HeLa cells and modulates parasite invasion. (**A**) Binding of rTcMVK on the HeLa cells. Paraformaldehyde fixed HeLa cells were incubated with increasing concentrations (nM) of rTcMVK (circle). As a His-tag negative control, HeLa cells were also incubated with rCaEnolase (square), a His-tag recombinant protein from *Candida albicans* that does not adhere to HeLa cells. This result is the mean of two independent experiments performed in triplicates ± standard deviation (SD). ***P < 0.001. Statistical analysis was performed by Two-way ANOVA method. (**B,C**) Opposite effects of rTcMVK in the cell invasion by EAs and MTs. rTcMVK enhances EA (**B**) but inhibits MT invasion (**C**). Giemsa staining of HeLa cells treated for 1 h with 300 nM of rTcMVK and incubated with EAs or MTs for 2 h. The multiplicity of infection was 10:1 or 20:1 for EA and MT forms, respectively. The negative control was carried out in the absence of rTcMVK. X axis: rTcMVK treated (rTcMVK) and non-treated (Ct) groups; Y axis: percentage of internalized parasites. The data correspond to the mean of six experiments performed in triplicates ± SD. *P < 0.05 and ***P < 0.001. Statistical analysis was performed by Student t test method.

Previous observations have indicated that *T. cruzi* proteins involved in host cell interactions usually bind to the host cell surface in a dose-dependent manner^22^,^29^. Adhesion assays using rTcMVK and fixed HeLa cells showed a non-saturable dose-dependent increase of bound enzyme to HeLa cell surface (Fig. 4A). The specificity of the assay was confirmed using a recombinant enolase of *Candida albicans* which did not bind to HeLa cells (Fig. 4A). To determine if rTcMVK could modulate *T. cruzi* internalization, HeLa cells were treated for 1 h with 300 nM (lowest concentration tested of rTcMVK that bound to HeLa cells), washed and incubated with either EAs or MTs of G strain for 2 h and the number of internalized parasites counted. TcMVK plays a role in parasite cell invasion but it has essentially opposite effects in EA and MT forms. The invasion of HeLa cells by EAs was significantly increased (Fig. 4B) whereas it was significantly inhibited in MTs (Fig. 4B). Even when rTcMVK was used at concentrations up to 1200 nM, no additional effects were detected (not shown). Additionally, invasion assays performed in the presence of anti-TcMVK resulted in opposite effects to those described above (MTs invasion increased, EAs decreased), further evidencing that the enzyme modulates parasite invasion (Figure SM2).

Control experiments showed that enolase, even at higher concentrations did not interfere in the parasite invasion rate (Figure SM3).

#### Signaling pathways activated by TcMVK

**Figure 5:**
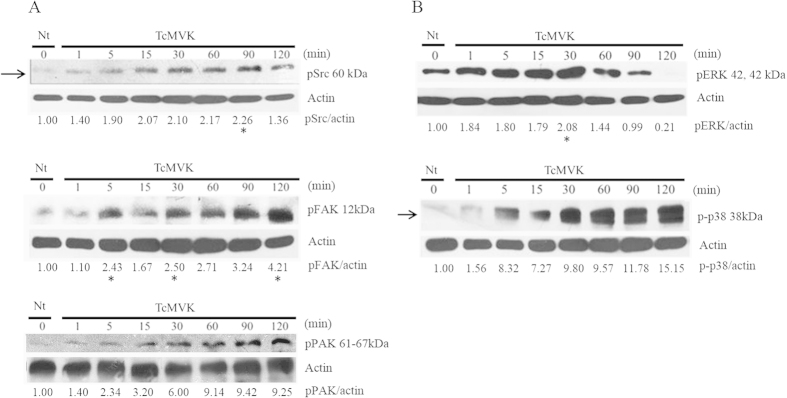
TcMVK induces phosphorylation of HeLa cell cytoskeletal modulators. (**A**) rTcMVK triggered time-dependent phosphorylation of Src (arrow), reaching a maximum phosphorylation at 90 min (asterisks). FAK displays increased TcMVK-dependent phosphorylation, noticeable at 5, 30 and 120 min (asterisks). PAK activation time-dependent increases up to 120 minutes. (**B**) rTcMVK induced the start of phosphorylation of MAPK components, ERK and p38, at 1 and 5 min, respectively. ERK displayed maximum activation at 30 min (asterisk), decreasing at 60 min and almost disappearing after 120 min. In contrast, p38 activation showed a time-dependent increase up to 120 min. Numbers under actin bands refer to the densitometric quantitation: protein/actin. Nt: negative control of cells without rTcMVK incubation. Arrows: time dependent increase; asterisks: phosphorylation peaks. Anti-actin was used as loading control. Band densitometry measurements were performed using UVIBAND^®^, UVITEC. Blots are representative of five independent experiments.

Considering that TcMVK is secreted into extracellular medium we hypothesized that this protein may interfere with different host cell signaling pathways such as cytoskeletal remodeling and cell migration thereby assisting in parasite invasion. Exposing HeLa cells to rTcMVK for various periods of time revealed that TcMVK induced a time-dependent activation of Src (Y416), a protein associated with actin microfilament rearrangement^30^,^31^,^32^, FAK (Y397) and PAK (S144), a protein known to be regulated by Rac1, subsequently modulating actin cytoskeleton reorganization, lamellipodium formation^33^,^34^ (Fig. 5A).

Another signaling cascade associated with cytoskeletal rearrangement is the mitogen-activated protein kinase (MAPK) pathway^30^,^35^. Incubation with rTcMVK for varying time periods induced activation of the MAPK components, ERK1/2 (T202/Y204), at 1 min, reaching a maximum at 30 min, and p38 (T180/Y182) in a gradual manner (Fig. 5B). All observed phosphorylated residues are implicated in protein activation.

## Discussion

*T. cruzi* host cell invasion is triggered partially by binding of membrane molecules of both cells responsible for mutual and specific signaling leading to parasite internalization^2^,^36^. In addition to surface molecules, *T. cruzi* forms can also secrete factors that interfere with host signaling to promote or inhibit parasite invasion^22^,^37^,^38^. In this study we found that *T. cruzi* MVK, an ergosterol biosynthesis essential enzyme, is secreted to extracellular medium and modulates host cell signaling during parasite invasion.

Using 3D molecular modeling and molecular mechanics we have evidence that TcMVK maintained structural requirements for substrate coupling, despite nucleotide and/or amino acid differences comparing to the others crystallized MVK (Fig. 2) (*L. major, T. brucei*^28^). *In vitro* assays using synthetic mevalonic acid confirmed enzymatic activity of recombinant TcMVK corroborating modelling data. Together these results were crucial to support the use of recombinant TcMVK in further biological observations – invasion and signaling assays.

MVK is located in peroxisomes in mammals and other eukaryotes^39^,^40^. Accordingly, in *L. major* and *T. brucei* MVK is confined to glycosomes, structures related to peroxisomes^8^. While *L. major* and *T. brucei* MVKs displayed complete colocalization to glycosomes^8^, our findings indicated that TcMVK partially colocalized with aldolase suggesting that TcMVK may also be located in other intracellular compartments. The fact that TcMVK is not present in mitochondria, acidic compartments and endoplasmic reticulum may indicate the participation of intracellular compartments related to non-canonical secretory pathways, such as microvesicules in TcMVK secretion^41^.

*T. cruzi* secretome analysis indicated that only approximately 9% of the 367 identified proteins have a signal sequence and are predicted to be secreted by classical pathways while 48% of them would be secreted by nonclassical pathways^41^. Secretome analysis revealed that *L. donovani* secretes several proteins classically involved in diverse intracellular functions, including metabolic pathways, protein folding, regulation or biosynthetic processes and RNA metabolism^42^,^43^. In addition, *T. brucei* and *T. cruzi* secrete proteins classically involved in cell cycling, catabolic process, cell signaling, transporting and protein synthesis^44^. Interestingly, several secreted proteins may also be involved in parasite virulence, facilitating host cell internalization^41^,^42^. Finally, enolase, a key glycolysis enzyme found in *L. donovani, T. brucei* and *T. cruzi* secretomes^41^,^42^,^44^ has been reported to be a possible important factor in trypanosome virulence^43^.

In the present study, rTcMVK is acknowledged to be highly active and to bind to HeLa cells similarly to previously observed to other *T. cruzi* proteins^22^,^45^. There is evidence that *T. cruzi* secretes proteins which may participate in host cell signaling pathways and modulate parasite invasion. For instance, the cysteine protease, cruzipain, is secreted^41^ and participates in parasite invasion^46^. Another *T. cruzi* protein, 21 kDa protein (P21) is secreted and interferes with HeLa cell invasion by EAs and MTs, acting as a phagocytosis inducer^22^,^47^. In addition, tissue culture trypomastigote forms (TCTs) secrete collagenase that loosens host cell extracellular matrix to achieve tissue migration^37^.

In addition to adhering to the host cell surface, we demonstrated that rTcMVK positively modulates EAs invasion whilst negatively regulating MTs invasion. A mechanistic link between cell signaling and cell invasion may be driven by the reorganization of cytoskeletal elements, particularly actin filaments, given that the uptake of EAs depends on actin cytoskeleton^48^,^49^.

Accordingly, we observed that rTcMVK can activate the actin related kinases Src/FAK and PAK and the MAPKs, ERK and p38^32^,^33^,^50^. Upon phosphorylation by integrins, Src associates and activates FAK to regulate several actin-related processes, including phosphorylation of key substrates such as paxillin and cortactin^32^,^51^,^52^,^53^,^54^,^55^. It has also been shown that activation of host cell Src and FAK is correlated to the actin dependent internalization of bacterial pathogens^56^,^57^,^58^,^59^. The serine-threonine kinase, PAK, acts as an effector of Rac1 and other GTPases^60^,^61^ and previous results from the author’s group demonstrate that cells expressing constitutively active Rac1 are more susceptible to EAs invasion^62^. Due to the close relation of Rac1 to PAK activation and consequent actin cytoskeleton rearrangements^33^,^63^, the increased PAK phosphorylation in HeLa cells treated with rTcMVK observed in the current study supports the notion that the secretion of this enzyme may trigger Rac1/PAK activation, leading to increased EAs internalization.

In addition, ERK and p38 were activated in HeLa cells incubated with rTcMVK. ERK mediates the activation of proteins that regulate microfilament remodeling such as calpain, FAK and cortactin^30^,^31^. Moreover, activation of ERK has also been associated with increased *T. cruzi* TCT invasion^64^. Despite this, the involvement of these proteins in actin related processes remains poorly characterized^35^ and their involvement in actin independent mechanisms cannot be ruled out. Collectively, these results suggest several mechanisms by which rTcMVK treatment of HeLa cells increases EAs uptake.

In contrast to EAs, rTcMVK inhibited MT invasion of HeLa cells. As MTs present distinct mechanisms to invade cells compared to EAs^49^,^62^, it was hypothesized that different signaling pathways are triggered during MT invasion in rTcMVK treated cells. Whilst it has been reported that HeLa cell invasion by MTs (G strain) is dependent upon the polymerization of actin filaments^65^, there is no relation to Src/FAK or PAK activation by rTcMVK observed in the present study. Conversely, activated ERK and p38 have been shown to prevent plasma membrane wounds caused by mechanical damage or bacterial toxins^66^,^67^,^68^. It is important to note the results of a study reporting that TCTs secrete (yet unknown) factors that decrease p38 and ERK activation^69^ hindering membrane wound repair, a mechanism known to be directly involved in parasite internalization^70^.

**Figure 6:**
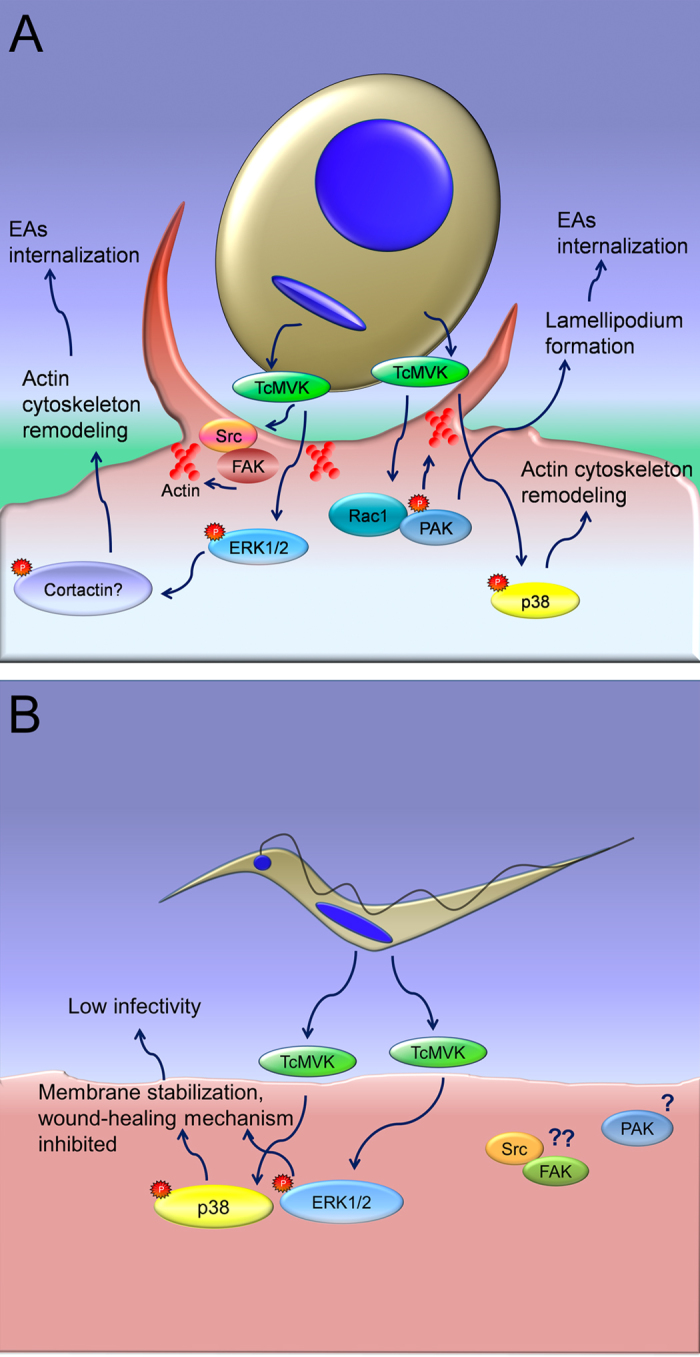
Schematic view of signaling pathways induced by TcMVK secretion. (**A**) EA invasion. TcMVK induces Src/FAK phosphorylation which is important to cytoskeleton remodeling which is also involved in invasion process of diverse intracellular pathogens. PAK, which is activated by Rac1, displays an important role in actin cytoskeleton rearrangements, culminating in *T. cruzi* EA invasion^62^. TcMVK induces ERK and p38, both involved in activation of pathways that culminates in cytoskeleton remodeling. Additionally, ERK mediates the activation of proteins that regulate microfilament remodeling such as calpain, FAK and cortactin^30^,^31^. (**B**) MT invasion. The inhibition of MT invasion in the presence of rTcMVK can be explained by ERK and p38 activation which prevent the mechanism of membrane repair (wound healing)^66^,^67^,^68^, described as essential to trypomastigote invasion^69^. Src/FAK and PAK activation by rTcMVK in MT invasion was not investigated in this study.

It is well known that MTs induce lysosomal exocytosis in HeLa cells^71^, a key event in membrane wound healing that drives parasite invasion^70^,^72^. Therefore, TcMVK activation of p38 and ERK in HeLa cells may help to stabilize membrane integrity and thus inhibit wound healing-driven internalization of parasites. A proposed model of HeLa signaling pathways induced by TcMVK secretion by both EAs and MTs is shown in Fig. 6.

As demonstrated in this study, TcMVK participates in parasite internalization, a process unrelated to the canonical activity of MVK. Similar to TcMVK, other proteins in several organisms have been shown to perform different functions aside from their traditional ones^73^. *Plasmodium spp.* enolase, *Trichomonas vaginalis* aldolase and GAPDH have been identified as adhesins to the host cell membrane^9^,^74^,^75^. In trypanosomatids, it has been described that *L. mexicana* enolase, in addition to its usual location in glycosomes, is found in the cytosol and the outer cell surface, assisting in plasminogen capture by the parasite^11^,^76^. Finally, *L. donovani* hexokinase, usually located in glycosomes, can be found at the flagellar pocket acting as a hemoglobin receptor, most likely participating in heme or iron acquisition^77^.

The results of the present study showed that the ergosterol biosynthesis enzyme, TcMVK, is secreted. It is hypothesized that TcMVK accesses the extracellular medium and diverse mechanisms such as microvesicle extrusions may also be involved, requiring further investigation. Finally, this kinase plays an unexpected role since it adheres to the cell surface, stimulating and regulating host cell responses towards EA and MT internalization, indicating that this kinase may be a new virulence factor in *T. cruzi*-host cell biology.

## Additional Information

**How to cite this article**: Ferreira, E. R. *et al*. Unique behavior of *Trypanosoma cruzi* mevalonate kinase: A conserved glycosomal enzyme involved in host cell invasion and signaling. *Sci. Rep.* **6**, 24610; doi: 10.1038/srep24610 (2016).

## Supplementary Material

Supplementary Information

## Figures and Tables

**Table 1:** rTcMVK specific activity compared to MVKs from different organisms available via BRENDA server ( http://www.brenda-enzymes.info/); MVK entry number: EC 2.7.1.36.

Organism	Specific kinase activity[μmol/min/mg]	Reference
*Methanocaldococcus jannaschii*	387.0	23
*Trypanosoma cruzi*	72.8	This study
*Homo sapiens*	37.0	78
*Rattus novergicus*	37.2	79
*Enterococcus faecalis*	24.0	80
*Staphylococcus aureus*	12.4	81
*Trypanosoma brucei*	Inactive	28
*Leishmania major*	Inactive	28
