# Supplementary material for: Unique behavior of Trypanosoma cruzi mevalonate kinase: A conserved glycosomal enzyme involved in host cell invasion and signaling
Source: Sci Rep. 2016 Apr 26;6:24610. doi: 10.1038/srep24610 (PMC4845012; doi:10.1038/srep24610)
Supplement: Supplementary Information [file srep24610-s1.doc]

**Supplementary material**

**Unique behavior of *Trypanosoma cruzi* mevalonate kinase: A conserved glycosomal enzyme involved in host cell invasion and signaling.**

**Éden Ramalho Ferreira^a^, Eduardo Horjales Reboredo^b^, Alexis Bonfim-Melo^a^, Cristian Cortez^a^, Claudio Vieira da Silva^c^, Michel De Groote^b^, Tiago José Paschoal Sobreira^d^, Mário Costa Cruz^a^, Fabio Mitsuo Lima^a^, Esteban Mauricio Cordero^a^, Nobuko Yoshida^a^, José Franco da Silveira^a^, Renato Arruda Mortara^a^, Diana Bahia^a, e#^**

Departamento de Microbiologia, Imunologia e Parasitologia, Escola Paulista de Medicina, Universidade Federal de São Paulo^a^, São Paulo, São Paulo, Brazil; Instituto de Física, USP, São Carlos, São Carlos, SP, Brazil^b^; Instituto de Ciências Biomédicas, Universidade Federal de Uberlândia, Uberlândia, MG, Brazil^c^; Laboratório Nacional de Biociências, Campinas, SP, Brazil^d^; Departamento de Biologia Geral, Instituto de Ciências Biológicas, Universidade Federal de Minas Gerais, Minas Gerais, Brazil^e^

#Address correspondence to Diana Bahia, [dianabahia@hotmail.com](mailto:dianabahia@hotmail.com). Departamento de Biologia Geral, Instituto de Ciências Biológicas, Universidade Federal de Minas Gerais. Av. Antônio Carlos 6627, Pampulha, 31270-910, Caixa Postal 486, Belo Horizonte, MG, Brazil.

**Figures:**

**SM1: Multiple alignment of genomic and cDNA sequences of TcMVK.**

**
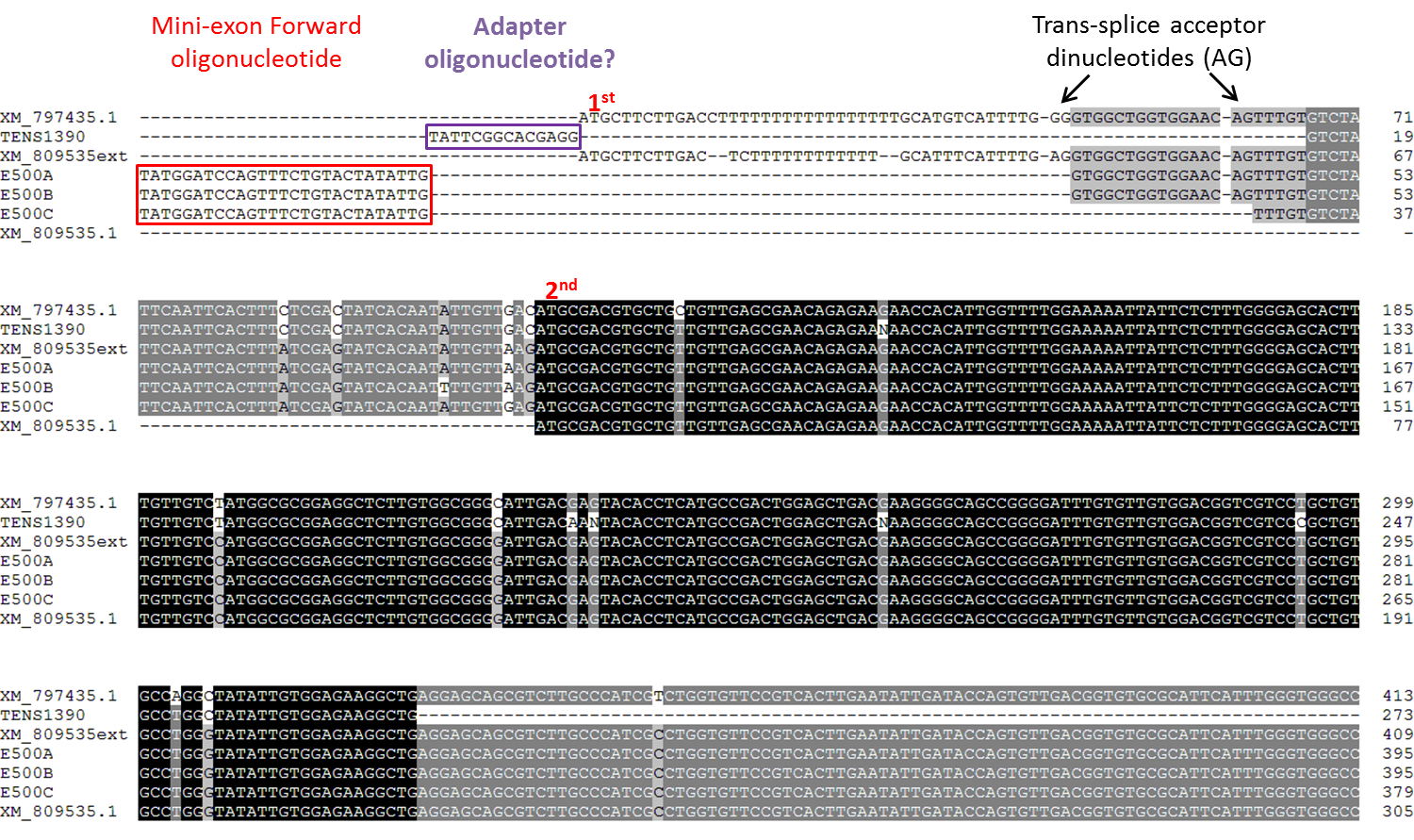
**

TcMVK copies isolated from genomic DNA or mRNA were arranged using Clustal W tool.  XM_797435.1 and XM_809535.1 are genomic sequences of MVK from T. cruzi clone CL-Brener. XM_809535ext is a 5' "in silico" extended copy of the XM_809535.1 sequence. TENS1390 is an EST from an epimastigote library (Clone CL-Brener) containing an adapter oligonucleotide (purple box). E500A, B and C are cDNAs amplified by RT-PCR from CL-Brener epimastigotes using a Mini-exon forward oligonucleotide (red box) and a MVK internal oligonucleotide (not shown). 1st, ATG star codon in the large MVK genomic copy; 2nd, ATG star codon in the MVK short copy. The arrows indicate the putative trans-splicing acceptor dinucleotides. The predicted signal peptide of the large copy of MVK is encoded by the region between both initiator ATG codons. Shade key: Black, 100 % identity, dark grey, 80% identity and light gray 60% identity

**SM2: Anti-TcMVK interferes in parasite invasion**


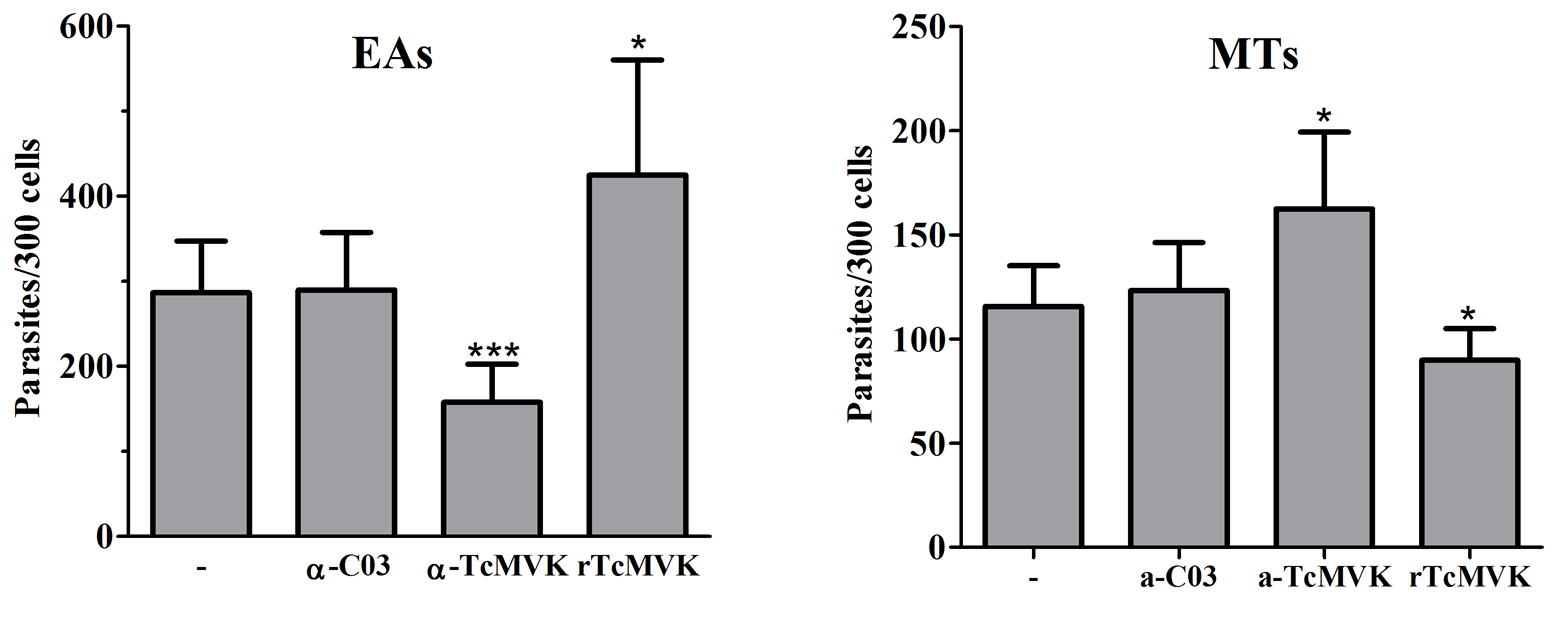


EAs and MTs were incubated with anti-TcMVK antibody (α-TcMVK), control antibody (α-C03), recombinant protein (rTcMVK) or untreated, washed, incubated with HeLa cells for 2 hours, stained with Giemsa and the number of intracellular parasites estimated under optical microscope. The data is representative of three independent experiments performed in duplicates ± standard deviation (SD). *: P< 0.05 and ***: P< 0.001. Statistical analysis was performed by Student t test method.

**SM3: HeLa non-adherent recombinant protein does not interfere in EAs cellular invasion**

**
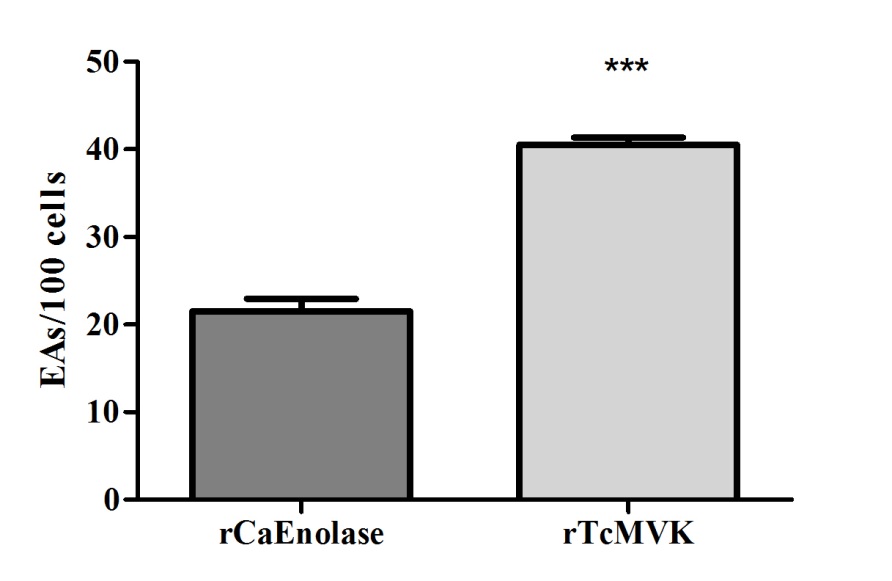
**

Recombinant *Candida albicans* enolase (300nM, rCaEnolase) does not influence EAs invasion rate, compared to rTcMVK incubation (300nM). The data is representative of three independent experiments performed in duplicates ± standard deviation (SD). ***: P< 0.001. Statistical analysis was performed by Student t test method.

**SM4: anti-TcMVK antibody specificity**


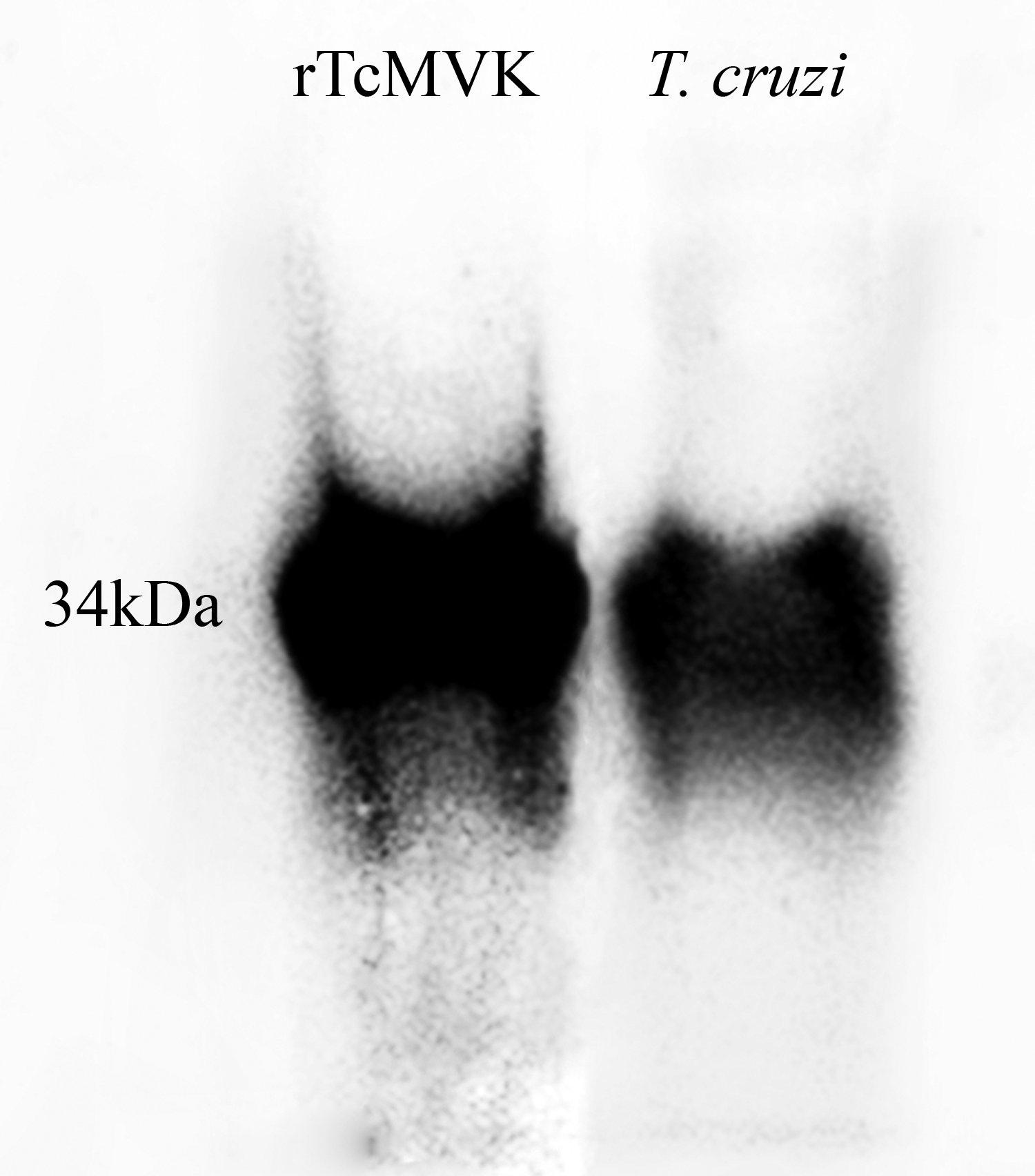


Western blotting assays demonstrated α-TcMVK antibody specificity to recombinant TcMVK (rTcMVK) and *T. cruzi* extracts. SDS-PAGE containing 1 µg of **rTcMVK** and 80 µg of ***T. cruzi*** extracts were transferred to a nitrocellulose membrane and incubated with α-TcMVK.

**SM5: Expression and purification of rTcMVK expressed in *E. coli.***


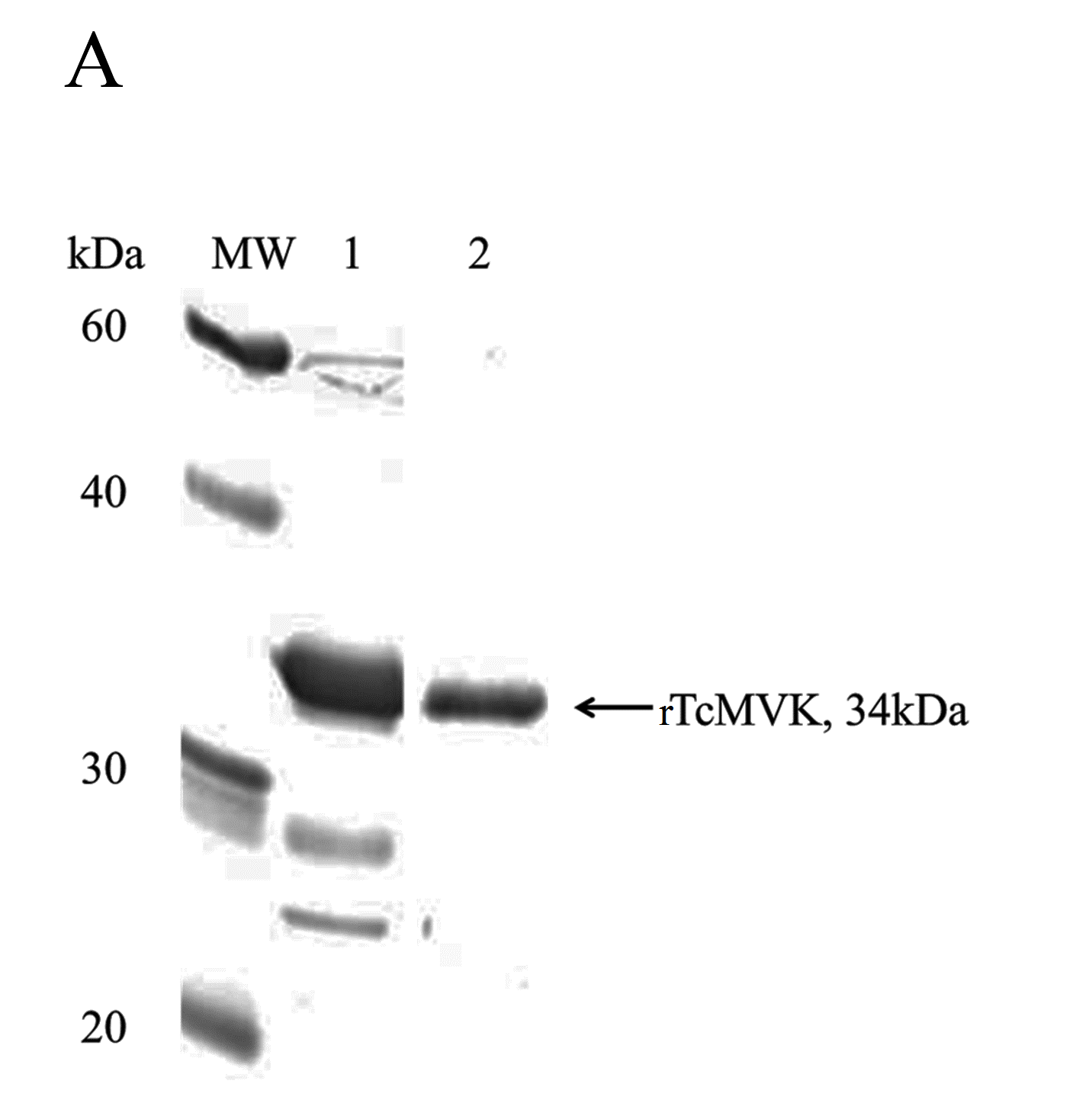


The recombinant TcMVK was purified using gel-filtration technique. Samples from bacterial extracts expressing TcMVK were loaded in on SDS-PAGE gels and stained with Coomassie. **MW:** Molecular weight markers; **1:** Bacterial extract expressing rTcMVK following elution from Ni-NTA column; **2:** Sample from the final purification step after gel-filtration of Ni-NTA purified extract.

**SM6: Purification of rTcMVK (monomer, dimer and tetramer) and measure of relative enzymatic activity**

**A**

**
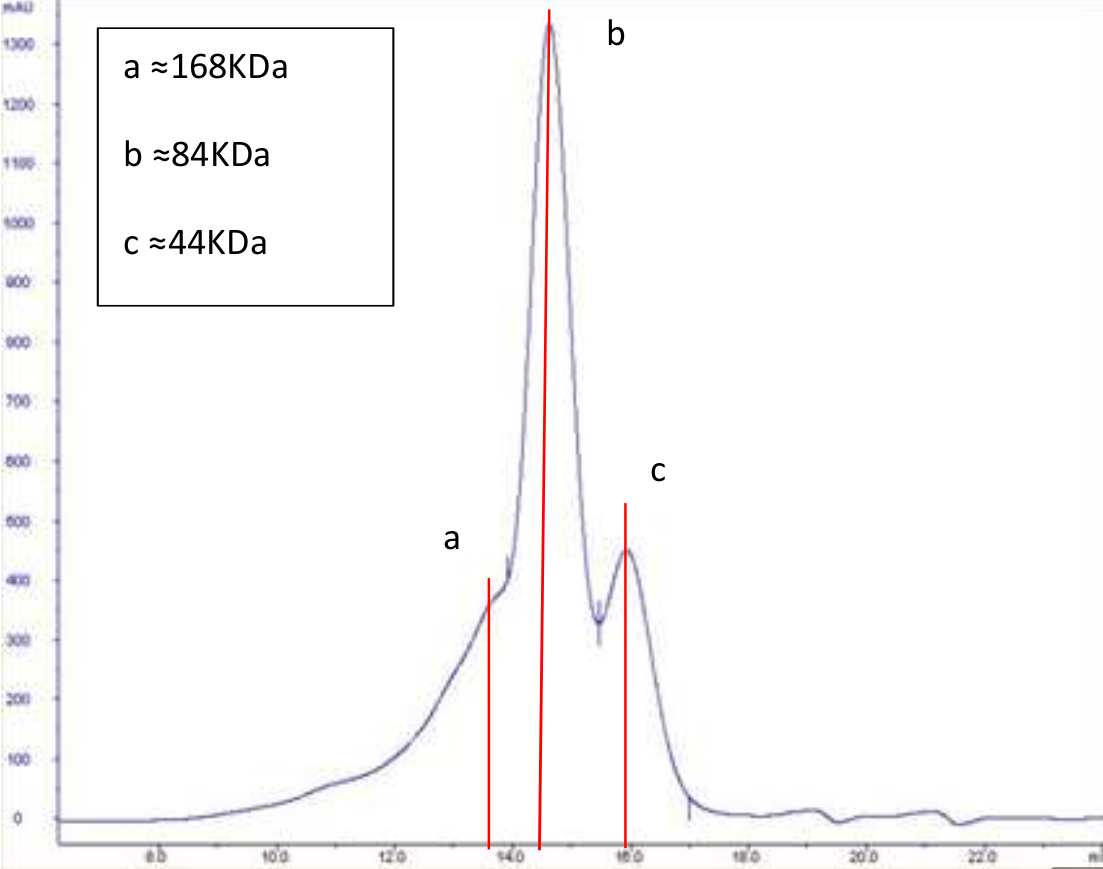
**

**B**

| **TcMVK** | **PH 9.0 μM/(min*mg)** |
| --- | --- |
| Monomeric sample (15 days stored) | 18.2 |
| Monomeric sample (fresh) | 13.0 |
| Dimeric sample  (15 days stored) | 19.7 |
| Dimeric sample (fresh) | 72.8 |

**The dimeric, not monomeric, TcMVK fraction has a high enzymatic activity. A:** Size exclusion chromatography was used to separate the oligomerization states. The peaks correspond to tetrameric (a), dimeric (b) and monomeric (c) fractions. **B:** Specific activity measurements of different TcMVK fractions.

**SM7: TcMVK does not colocalize with mitochondrial, acidic compartments, or endoplasmic reticulum markers.**

A

**
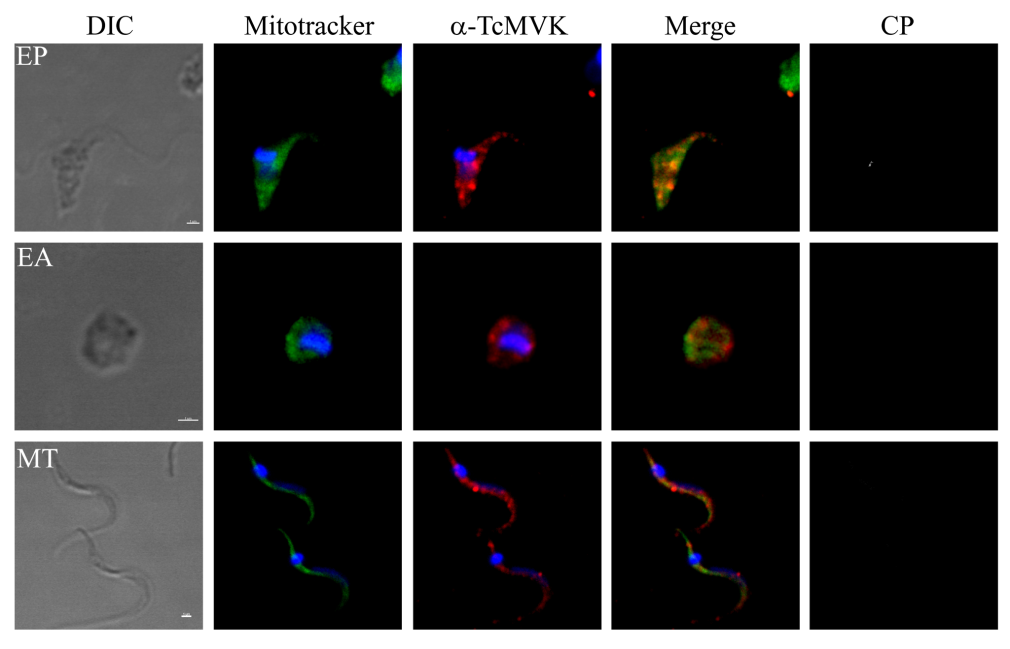
**

**B**

**
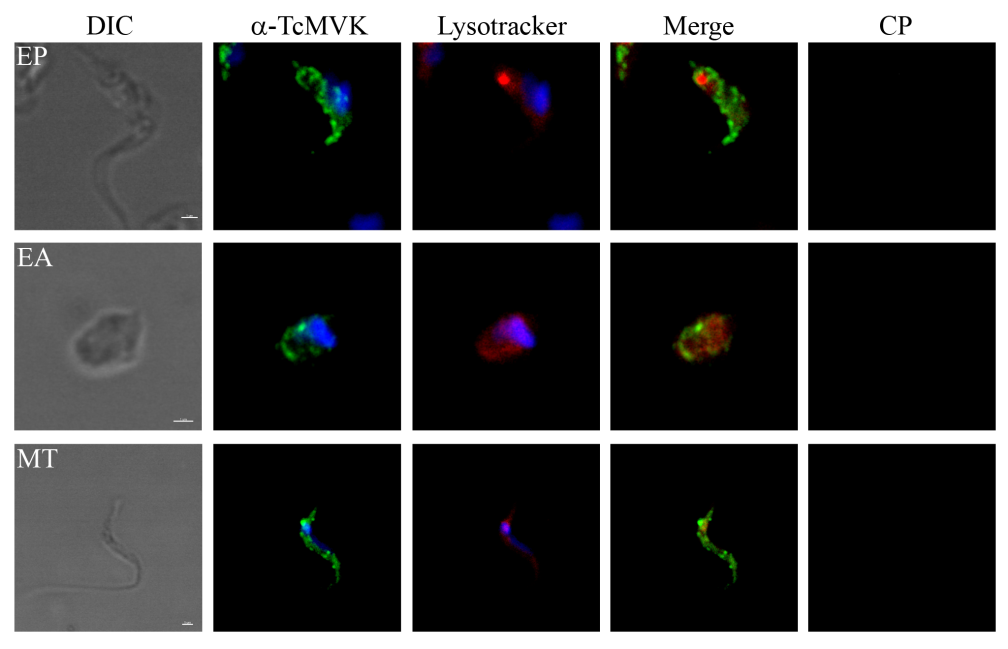
**

**C**

**
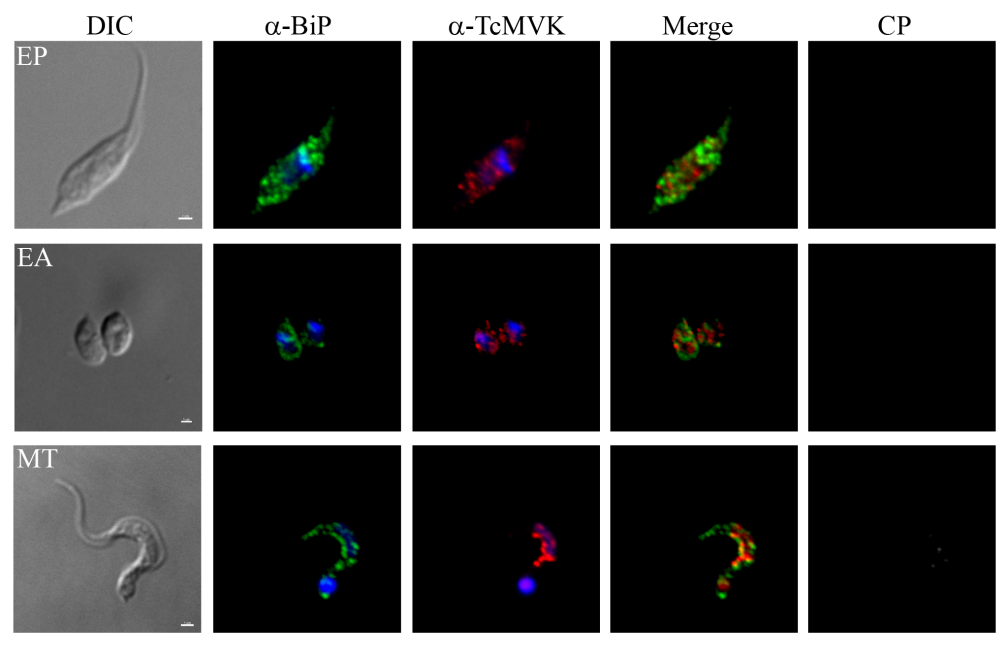
**

**TcMVK does not colocalize with mitochondrial, acidic compartments or endoplasmic reticulum (ER) markers**. Immunofluorescence images acquired with confocal microscope show that TcMVK does not colocalize with any of intracellular compartments markers used as shown by the absence of colocalized pixels (CP) in all *T. cruzi* evolutive forms evaluated (epimastigotes, EP; extracellular amastigotes, EA; metacyclic trypomastigotes, MT). **A:** mitotracker for mitochondria. **B:** lysotracker for acidic compartments. **C:** BiP as ER marker. Differential interference contrast (DIC), DAPI (blue). Images represent at least 100 parasites/group. Bar: 1 µm.
